# Unilateral Choroidal Granuloma and a Pupillary Abnormality in a Case of Miliary Tuberculosis: A Dilemma for the Physician

**DOI:** 10.7759/cureus.28713

**Published:** 2022-09-02

**Authors:** Vijaya Jojo, Poonam Singh, Rudra P Samanta, Reyaz Ahmad

**Affiliations:** 1 Ophthalmology, Tata Main Hospital, Jamshedpur, IND; 2 Pulmonology, Tata Main Hospital, Jamshedpur, IND; 3 Neurology, Tata Main Hospital, Jamshedpur, IND

**Keywords:** visual loss, miliary tuberculosis, afferent pupillary defect, choroidal tubercle, ethambutol toxicity

## Abstract

A young woman presented to the emergency with acute paraplegia and vision loss. She was diagnosed two months ago as a case of miliary tuberculosis with involvement of the chest and brain and therefore was on anti-tuberculosis treatment (ATT). She developed a decrease in vision and her treating physician suspecting optic neuropathy altered the regimen so as to omit Ethambutol and replaced it with Streptomycin. This treatment could not be continued with the advent of the COVID-19 pandemic as it required a hospital visit. On admission, she gave a history of inconsistent treatment and the ophthalmology evaluation showed decreased vision in the left eye, a relative afferent pupillary defect (RAPD), and a large solitary choroidal tubercle at the posterior pole of the same eye. The right eye was normal. On discussion with the treating physician, the standard four-drug ATT was reinstituted.

Through our case report, we wish to highlight a challenging situation wherein the vision loss and pupillary abnormality with a background of ATT led to the change of treatment that would have required either daily hospital visits or other arrangements to be made to provide the same at home. This modified regimen not only proved to be challenging for the patient and caregivers but also may have played a role in the newer onset of further complications secondary to an irregular treatment regime.

## Introduction

The incidence of miliary tuberculosis is two percent of all new cases of tuberculosis as reported by Sharma [1 ]. The ophthalmic involvement in this condition is so prominent that literature evidence suggests it to be a pointer in the diagnosis of miliary tuberculosis itself [2].

The treatment to date is an accepted regime of anti-tuberculous treatment (ATT) composed of a standard four drug regime in a fixed dose form and composed of Isoniazid, Ethambutol, Pyrazinamide and Rifampicin; though the duration may vary depending upon the diagnosis as a baseline. However, it is well known that one of the prominent side effects of ATT is visual loss secondary to Ethambutol toxicity. Ethambutol has been documented to cause partial or profound visual loss as early as two months [3]. The optic nerve is affected herein with the variable affliction of visual functions and may be partially reversible. Classically in established cases, there is the presence of an afferent pupillary defect accompanying the vision loss, usually in both eyes however may be asymmetrical or in close continuity.

## Case presentation

**Figure 1:**
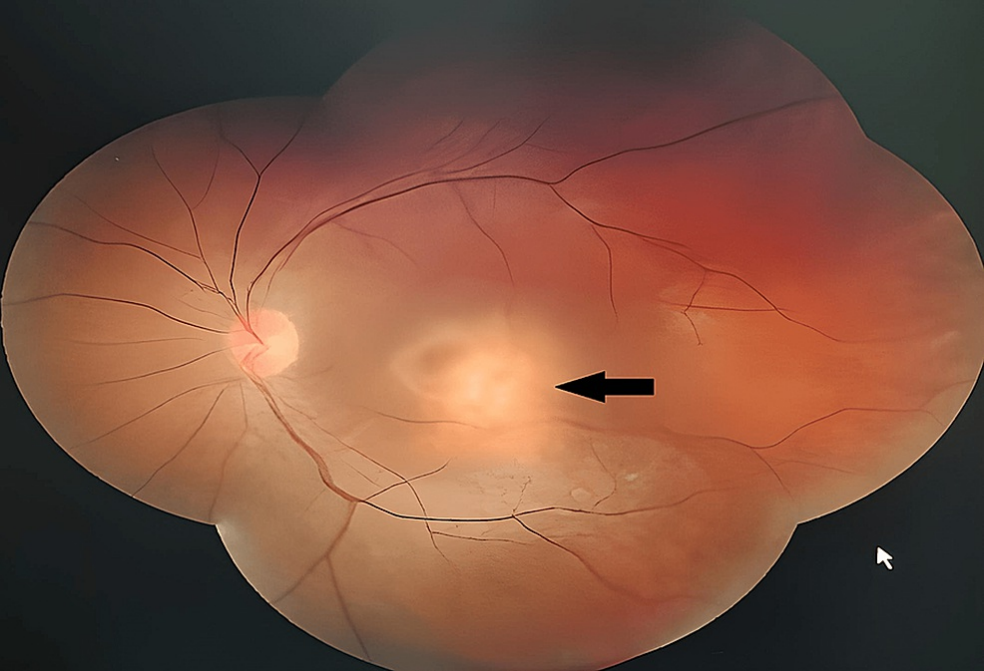
Left eye – solitary choroidal tubercle occupying the posterior pole

A 33-year-old software professional presented to the hospital emergency services with acute paraplegia. Two months prior to this she was diagnosed at her workplace hospital to have miliary tuberculosis, for which the Isoniazid, Ethambutol, Rifampicin, and Pyrazinamide (HRZE) regimen was started. Two weeks later, she developed progressive unilateral loss of vision, and the treating physician suspecting Ethambutol toxicity stopped it replacing it with an injection of Streptomycin. In the interim the COVID-19 pandemic created restrictions on accessing health care facilities inadvertently interrupting her treatment. She was shifted from her workplace hospital to our hospital; however, by then she had developed acute paraplegia and a further decrease in vision in the left eye. On admission, she was placed under treatment in the Neurology department and sought the opinion of the Pulmonologist and Ophthalmologist. The Ophthalmology evaluation noted her visual acuity to be 6/6 on the right eye and 6/60 on the left eye along with a normal pupillary response on the right eye, and a relative afferent pupillary defect (RAPD) on the left respectively. Anterior segment evaluation was normal; however, a dilated fundus examination showed the presence of a solitary elevated choroidal lesion of around four-to-five disc diameters in size occupying the posterior pole of the left eye (Figure 1). The right eye was noted as normal.

**Figure 2:**
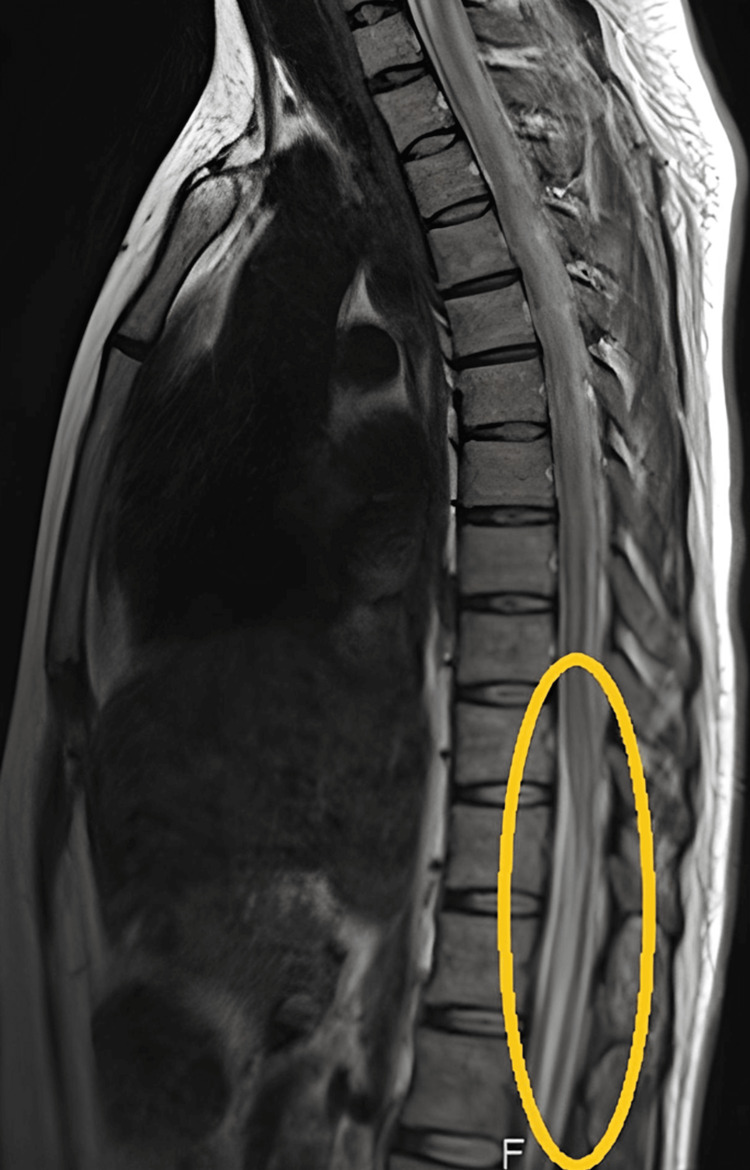
MRI spine showing transverse myelitis with mild disc desiccation at L5-SI level

A neurological and magnetic resonance image (MRI) evaluation showed her paraplegia likely to be transverse myelitis (Figure 2).

**Figure 3:**
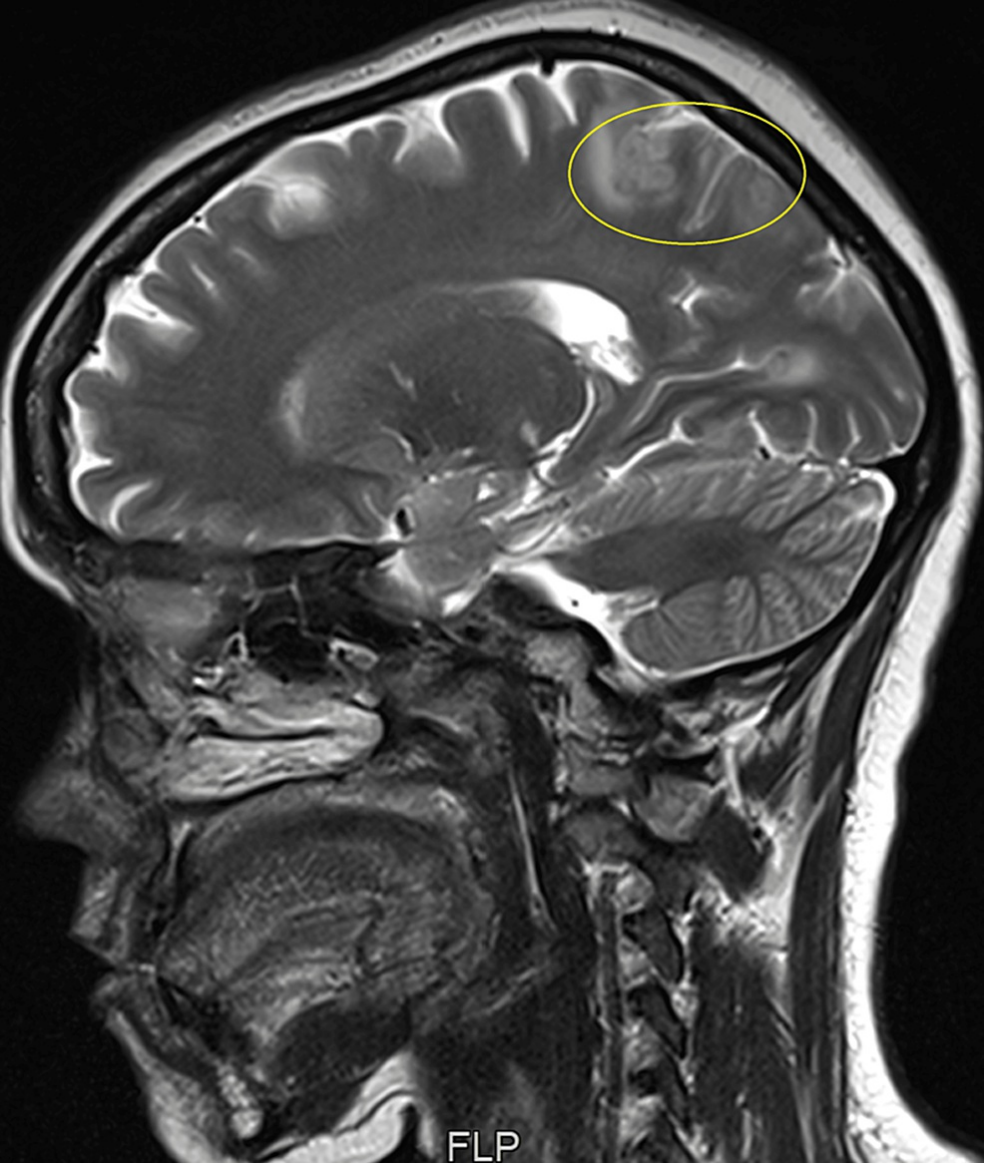
MRI brain sagittal section – multiple choroidal tubercles near corpus callosum

Apart from this, there was also disc desiccation at the L5-S1 level. The MRI brain showed multiple tuberculomas affecting the cerebral hemispheres near the Corpus Callosum (Figure 3).

**Figure 4:**
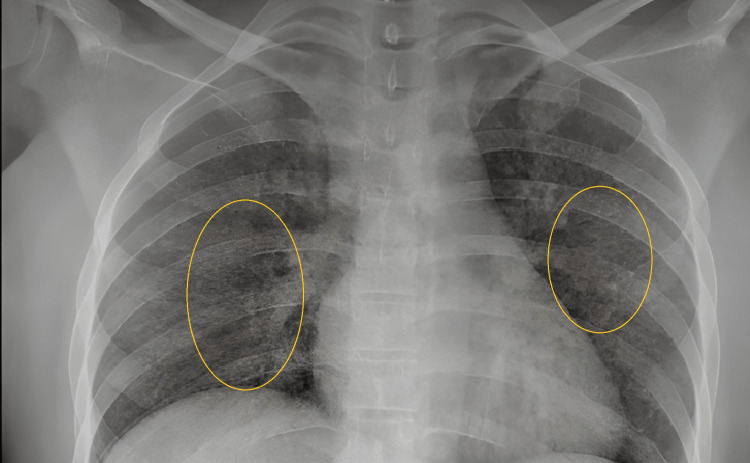
Chest x-ray – miliary mottling of both lung fields

Her chest x-ray was had significant miliary mottling of lung fields suggestive of miliary form of tuberculosis (Figure 4).

She was then under the combined care of the Neurologist, Pulmonologist, and Ophthalmologist and it was concluded that there was no evidence of ethambutol induced optic nerve toxicity however there was an urgent need to restart her on the treatment to check the further spread of the disease.

Ethambutol was restarted as per standard protocol with instructions given to the patient to report if any worsening or new ocular symptoms arose. She was then discharged on the same medications with follow-up advice. However, due to COVID-19 restriction and mobility issues, she is yet to be reviewed in the outpatient department however a telephonic conversation with the patient suggested her to be improving and that no further visual loss has occurred to date.

## Discussion

Miliary tuberculosis originated from the name Miliaris -Latin for millet-like. It is diagnosed as the involvement of at least two non-contiguous organs or infection of the blood, bone marrow, or liver with miliary mottling of the chest x-ray being pathognomonic as in our patient. The presence of choroidal tubercles is a hallmark of this clinical condition; however, diagnosing the same can be a challenge in routine medical practice [4].

In India, currently, the number of chest specialist reported by various undocumented sources are low at approximately 2,500, which would require patients to be under the care of General physicians; also currently the number of Ophthalmologists is scarce at one per 100,000 [5]. These figures could further mean that not every patient diagnosed with tuberculosis would have easy access to a Chest physician or an Ophthalmologist but are more likely to be under the care of primary care physicians. These patients would thereon be standard ATT and also likely to be followed up at the same medical facility. Under such situations, the development of vision loss is almost always thought to be secondary to ethambutol toxicity; this becomes more difficult in the presence of an obvious RAPD. This RAPD, however, has also been noted to occur in macular lesions, especially if there is asymmetry of involvement as noted in our case [6].

Most developing countries like ours could face a similar situation and the ensuing confusion could lead to incomplete treatments, this not only causing the further spread of the disease in the community but also leading to the emergence of multidrug-resistant tuberculosis variants [7]. Through our case, we wish to highlight that every afferent pupillary defect does not necessarily have to be related to an optic nerve lesion and the enormity of vision loss can cause an abnormal pupillary response. In fact, it has been advised that in cases where unilateral vision loss is noted alternate diagnoses must be considered before the drug is considered as the being the primary culprit [8].

Given the current healthcare provision in India and other such regions in the world, we also wish to reiterate to all Primary healthcare professionals to look at a clinical situation in its entirety rather than going by isolated clinical signs. This is more so when deciding on changes in standard treatment regimes, as the effects of these decisions may be profound with far-reaching consequences.

## Conclusions

Our case report wishes to call attention to a clinical situation in which primary care clinicians could misinterpret some typical signs, such as in our patient leading to a complete shift in a treatment regimen. In situations especially in developing countries where specialist opinion is not easily accessible at short notice, such circumstances might very well arise, hence keeping a broader perspective would lead Primary care and general practitioners toward the correct treatment modality. A consolidated multidisciplinary approach for this patient made patient care more comfortable and accurate, particularly for our patient, who was already physically incapacitated, and the ongoing COVID-19 situation made hospital visits more challenging.
